# Torsobarography: Intra-Observer Reliability Study of a Novel Posture Analysis Based on Pressure Distribution

**DOI:** 10.3390/s24030768

**Published:** 2024-01-24

**Authors:** Nico Stecher, Andreas Heinke, Arkadiusz Łukasz Żurawski, Maximilian Robert Harder, Paula Schumann, Thurid Jochim, Hagen Malberg

**Affiliations:** 1 Institute of Biomedical Engineering, Dresden University of Technology, 01307 Dresden, Germany; 2 Institute of Health Science, Collegium Medicum, Jan Kochanowski University, 25-369 Kielce, Poland

**Keywords:** posture analysis, pressure distribution, capacitive pressure sensor, topographic system

## Abstract

Postural deformities often manifest themselves in a sagittal imbalance and an asymmetric morphology of the torso. As a novel topographic method, torsobarography assesses the morphology of the back by analysing pressure distribution along the torso in a lying position. At torsobarography’s core is a capacitive pressure sensor array. To evaluate its feasibility as a diagnostic tool, the reproducibility of the system and extracted anatomical associated parameters were evaluated on 40 subjects. Landmarks and reference distances were identified within the pressure images. The examined parameters describe the shape of the spine, various structures of the trunk symmetry, such as the scapulae, and the pelvic posture. The results showed that the localisation of the different structures performs with a good (ICC > 0.75) to excellent (ICC > 0.90) reliability. In particular, parameters for approximating the sagittal spine shape were reliably reproduced (ICC > 0.83). Lower reliability was observed for asymmetry parameters, which can be related to the low variability within the subject group. Nonetheless, the reliability levels of selected parameters are comparable to commercial systems. This study demonstrates the substantial potential of torsobarography at its current stage for reliable posture analysis and may pave the way as an early detection system for postural deformities.

## 1. Introduction

Deformities of the spine are typically characterised by an asymmetrical morphology of the torso (e.g., in the case of adolescent idiopathic scoliosis, AIS [1,2]) and a sagittal imbalance [3]. The resulting surface characteristics serve as clinical symptoms for the early detection and progression assessment of postural disorders [4]. Pubertal growth often increases the progression [5]. As a consequence, postural disorders can manifest themselves in ways that require complex interventions, such as brace treatment, or even invasive surgical procedures [6]. Early detection of postural deformities, such as posture screening at schools, allows for early therapeutic intervention to reduce progression [7]. The posture of affected subjects is analysed manually by clinical inspection and functional tests according to several characteristics [8]. Objective devices, like the scoliometer, are employed for assessing posture [9]. However, these are limited to specific body segments. There is a need for reliable posture assessment tools that objectively assess the body segments as comprehensively as possible [10].

The gold standard for the diagnosis and assessment of spinal deformities is radiographic images (X-rays) of the torso. Depending on the initial diagnosis and clinical progression, X-rays may be taken multiple times a year [11]. The Cobb method is used to quantify and categorise lateral and sagittal curvatures of the spine in X-rays. In particular, computer-aided approaches to determine the Cobb angle achieve consistent and reliable results [12,13,14]. However, the high reliability during a radiological examination goes hand in hand with an increased radiation dose for the patient, which can consequently lead to long-term damage, such as cancer [15,16]. The current focus is dedicated to the reduction in use of X-rays, and the application and development of systems with a low radiation dose (e.g., EOS systems [17]) as well as radiation-free systems [11].

According to the SOSORT consensus statement [11], topographic systems may be utilised to screen and monitor scoliosis patients, reducing lifetime radiographs. Topographic systems are mainly camera-based and analyse the entire back as well as the pelvis. The Formetric 4D system (DIERS International GmbH, Schlangenbad, Germany), a commercial rasterstereographic system, is employed for the static assessment of spinal deformities. The system approximates the shape of the spine by analysing the surface gradients. The parameters characterise the frontal (e.g., scoliotic angle), sagittal (e.g., kyphotic angle) and transverse plane (e.g., surface rotation) of the approximated spine and pelvis. Studies have shown that several Formetric 4D parameters have a high intra- and inter-observer reliability (ICC > 0.75) [18,19,20,21]. Another example of a commercial system is the depth-camera-based Spine3D system (Sensor Medica, Guidonia Montecelio, Italy). Molinaro et al. investigated the reproducibility of the Spine3D system and identified a high intra-observer reliability [22]. The composition of Spine3D parameters includes features in the frontal (e.g., shoulder inclination), sagittal (e.g., lordotic angle) and transverse planes (e.g., pelvic torsion). Further topographic methods utilised for analysing spinal deformities include moiré topography [23], thermography [24] and photogrammetry [25]. All the named systems are stationary, require a suitable installation space and need professional instruction prior to their initial use. In addition, commercially available systems require the back to be fully exposed, which reduces compliance [9].

This paper proposes a novel topographic system (called torsobarography), which analyses the dorsal pressure distribution of the human torso in the lying position. The torsobarography method was developed at the Institute of Biomedical Engineering at TU Dresden in the 2010s [26]. In a biomedical context, the analysis of pressure distribution is already being investigated for orthostatic posture in asymmetrical sports [27], gait analysis [28], sleep posture classification [29] and even for the prevention of pressure ulcers [30]. Torsobarography relates the anatomical back morphology to the morphology of the dorsal pressure image. Gravimetric transmitted structures are identified and parameterised regarding their degree and symmetry.

Systems that are suitable for regular screening (e.g., during school PE lessons) should be versatile to deploy. The system utilises a flexible pressure sensor mat that is easily transported and does not necessitate recalibration upon location change, considerably simplifying its application. Commercial topographic systems can only employ the back surface to reconstruct the posture and the shape of the spine. Meanwhile, torsobarography offers insights into the mass distribution of the trunk alongside surface information, potentially unlocking deeper knowledge of trunk deformities. Furthermore, the measurements are conducted while subjects are clothed, thereby mitigating any adverse effects on compliance. The development of new tools for analysing posture provides an opportunity to improve the understanding of postural deformities. However, before the diagnostic application of torsobarography as a posture analysis system can be considered, it is necessary to evaluate the reliability of the system to ensure the scientific acceptance of the collected data. Thus, this study focuses on the intra-observer reliability of the extracted torsobarography parameters to assess the reproducibility of the measurements.

## 2. Materials and Methods

### 2.1. Participants

Forty subjects participated in the study, 20 females and 20 males, with an age distribution of 27.8 ± 4 years (range: 21–38 years), height of 174 ± 9 cm (range: 155–195 cm), and weight of 72 ± 13.2 kg (range: 51.3–111.4 kg). Subjects were asked about any history of postural disorders: two subjects reported that scoliosis had been diagnosed in the past, had been treated but not surgically corrected and was still present. The inclusion criteria for participation were the absence of musculoskeletal injuries and the ability of the subjects to perform the standardised measurement procedure (the image in Section 2.3). Subjects wore tight-fitting clothing (without bras or belts) during the measurement to prevent folds from appearing in the pressure image.

### 2.2. Instrumentation

**Figure 1:**
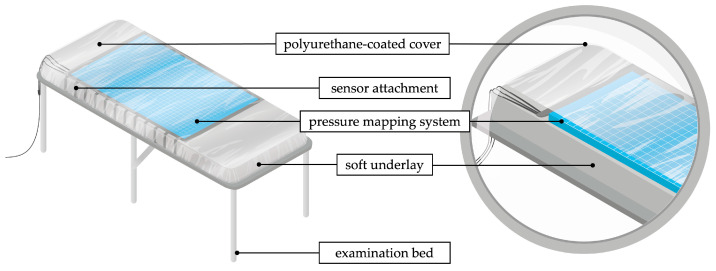
Schematic measurement setup of the torsobarography.

The dorsal pressure distribution of the torso in the lying position was measured with a high-resolution pressure mapping system (LX100:100.160.05, XSENSOR Technology Corporation, Calgary, AB, Canada). The sensor mat comprises a flexible, capacitive pressure sensor array consisting of 100 × 160 sensors on an area of 50.8 cm × 81.2 cm. The pressure range is calibrated for 0.07–2.7 $N/{\mathrm{cm}}^{2}$ with an accuracy of ± 5%. The sensor mat was attached to an examination bed (Figure 1). A soft underlay ensured the contact of the sensor mat over the entire length of the torso. The underlay consisted of a 6 cm thick self-inflating pad (BLACK CREVICE, Connective Sport Handels.m.b.H., Vöcklamarkt, Austria). A polyurethane-coated cover (Spannbezug special^®^, Dr. Güstel Waschfaserlaken GmbH & Co. KG, Pinneberg, Germany) was stretched over the system for hygiene and to protect the sensor mat from shear stress. Data acquisition, processing and storage were performed with a notebook connected via USB. Matlab R2022a was used for data processing.

### 2.3. Measurement Procedure

**Figure 2:**
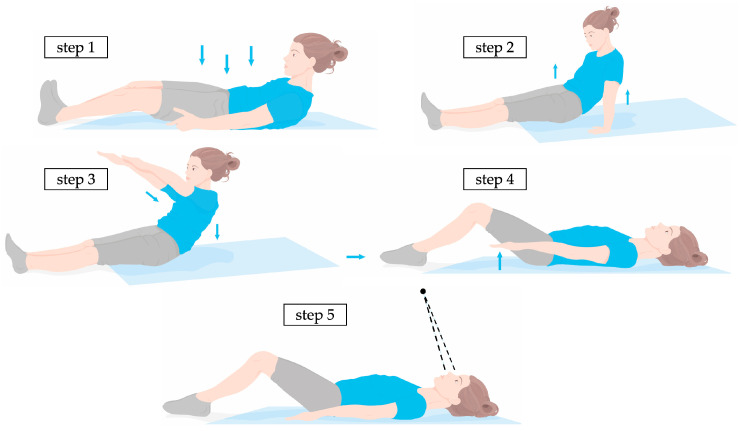
Schematic of the measurement procedure for the positioning of a subject; step 1: positioning on the sensor and ensuring that the entire torso is detected; step 2: lifting the pelvis; step 3: lowering the torso with arms stretched; step 4: bending the knees by approx. 100°; step 5: palms facing downwards and looking upwards.

A standardised measurement procedure was used to position the subject. The subject first laid down in the centre of the mat as shown in Figure 2. It is crucial that the entire torso from the top of the shoulder to the bottom of the buttocks is detected by the sensor. In the second step, the subject straightened up again and lifted the pelvis to neutralise potential tensions. In the third step, the subject stretched the arms out shoulder-width apart and slowly lowered the upper body onto the mat surface. In the fourth step, subjects were directed to flex their knees to form an angle of approximately 100° at the knee joints. This position diminished lumbar lordosis and improved the sensor’s detection of the lumbar region. The arms are placed next to the torso with the palms facing down, a few centimetres apart. In the fifth step, the subject looked up towards a fixed point on the ceiling, whereupon a measurement was initiated.

The measurement duration was set to 10 s at a sample rate of 10 Hz. Each subject was measured a total of ten times in accordance with the standardised measurement procedure. The subjects rested in a standing position for one to two minutes between each measurement. Subsequently, the subjects were instructed to reposition on the mat. This method guaranteed a unique orientation of the participant on the sensor for each measurement, promoting the independence of the recorded data.

### 2.4. Data Preprocessing

For the analysis of the pressure distribution, a single frame $I_{\mathrm{sel}}\left(j,i\right)$ was selected from each measurement to reduce the influence of movements and breathing. An average pressure intensity value was calculated for each of the recorded frames. From this, the medial frame $I_{\mathrm{sel}}\left(j,i\right)$ was selected. 

The selected frame $I_{\mathrm{sel}}\left(j,i\right)$ was filtered with a median filter (15 × 5) and a gaussian filter (3 × 3, σ = 3). The median filter reduced high-frequency local artifacts, which were caused by the folds of the sensor mat or the subject’s clothing. The Gaussian filter smoothed edges induced by the sensor resolution and by the median filter. The preprocessed pressure image I(j,i) resulted in a torso imprint with a smooth, continuous surface. The filtering applied to I(j,i) resulted in a distortion of high-frequency structures, such as peaks intensified by the scapulae, affecting both their intensity and position. Based on this, a second pressure image $I_{s}\left(j,i\right)$ was generated, whereby $I_{\mathrm{sel}}\left(j,i\right)$ was filtered exclusively with a 5 × 1 dimensioned median filter. Subsequent processing steps that transformed the pressure image were applied for I(j,i) as well as for $I_{s}\left(j,i\right)$.

**Figure 3:**
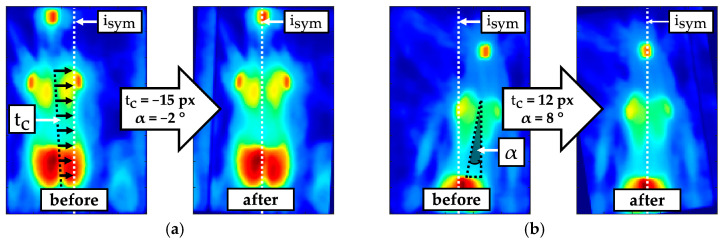
Example of comparison before and after derotating by angle α and centring by translation $t_{c}$ to align the symmetry axis of the torso imprint towards the centre of the pressure image $i_{\mathrm{sym}}$; (**a**) Predominant translation; (**b**) high translation and rotation.

A transformation for derotating and centring was applied with the aim of shifting the mirror symmetry axis of the torso imprint to the horizontal centre of the pressure image $i_{\mathrm{sym}}=50$ (Figure 3). The result was an almost symmetrically oriented torso imprint, which simplifies the detection of symmetry deviations. Values for the translation t and rotation α of the torso imprint were determined iteratively by minimising the quality criterion $\xi_{\alpha ,t}$ (1) within an optimisation function (1).

(1) $$\underset{\alpha ,t}{\min}\xi_{\alpha ,t}\;\mathrm{for}\;\forall \left\{\alpha \;\in Z\right|\;-15\;{}^{\circ}\;\leq \;\alpha \;\leq \;15\;{}^{\circ}\}\;\mathrm{and}\;\forall \left\{t\;\in Z\right|\;-30\;\mathrm{px}\;\leq \;t\;\leq \;30\;\mathrm{px}\}\;$$

The quality criterion $\xi_{\alpha ,t}$ (2) describes the sum of the absolute differences (SAD) between the mirrored α-rotated pressure image $I_{\alpha }^{*}\left(j,i\right)$ and the α-t-transformed pressure image $I_{\alpha ,t}\left(j,i\right)$. To obtain the translation $t_{c}$ that centres the torso imprint, the determined translation t must be halved.

(2) $$\xi_{\alpha ,t}=\sum_{j=1}^{160}\sum_{i=1}^{100}\left|I_{\alpha ,t}(j,i)\;-I_{\alpha }^{*}\left(j,i\right)\right|$$

**Figure 4:**
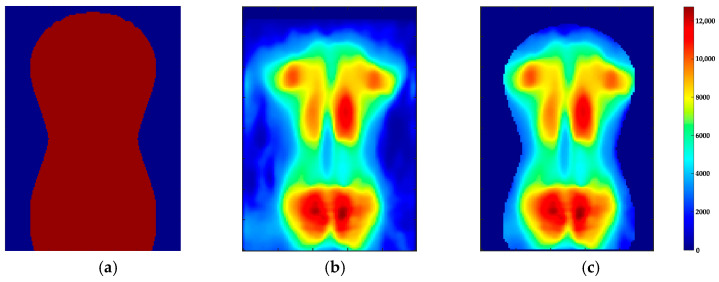
Segmentation of the pressure image I(j,i) with the mask B(j,i) to obtain the $I_{\mathrm{roi}}\left(j,i\right).$ (**a**) B(j,i); (**b**) pressure image; (**c**) ROI.

The lower edge of the torso imprint was shifted to row j=160. Pressure intensities of structures that cannot be assigned to the torso and shoulders, such as the arms or the head, as well as other artefacts caused by folds of the mats, should have minor impact on the parameter extraction. Therefore, the torso imprint was segmented into a region of interest (ROI) using masking. The mask B(j,i) (3) according to Figure 4 was scaled with the factors $s_{j}$ and $s_{i}$.

(3) $$B^{’}(j’,i’)=f_{\mathrm{scale}}\left(B(j,i),s_{j},s_{i}\right)$$

The dimension of $B^{’}(j’,i’)$ was adjusted to the dimension of I(j,i) by filling missing values with zero. The identification of optimal scaling factors ($s_{j}$, $s_{i}$) was conducted by minimising the energy function $E_{\mathrm{roi}}$ (4). If the pressure intensities inside ($j,i\in \;\Omega_{i}$) and outside of the mask ($j,i\in \;\Omega_{o}$) match the corresponding mean values (${\overset{-}{I}}_{\Omega_{i}}$, ${\overset{-}{I}}_{\Omega_{o}}$), $E_{\mathrm{roi}}$ reaches a local minimum. The mask scaled by the optimal factors was multiplied by the pressure image I(j,i), which resulted in the ROI $I_{\mathrm{roi}}\left(j,i\right)$.

(4) $$E_{\mathrm{roi}}=\underset{\forall s_{j},\;s_{i}}{\mathrm{argmin}}\left(\sum_{j,i\;\in \Omega_{i}}\left(I\left(j,i\right)-{\overset{-}{I}}_{\Omega_{i}}\right)+\sum_{j,i\;\in \Omega_{o}}\left(I\left(j,i\right)-{\overset{-}{I}}_{\Omega_{o}}\right)\right)$$

### 2.5. Landmark Identification

First, the landmark for the start of the torso $j_{\mathrm{ts}}$ was localised according to Equation (5). The threshold value (tv) was set to 7000, which is equal to the empirically determined maximum noise amplitude.

(5) $$j_{\mathrm{ts}}=\underset{\forall \;j}{\min}\left(\;j\;|\;\sum_{i=1}^{100}I_{\mathrm{roi}}\left(j,i\right)>\;\mathrm{tv}\;\right)$$

**Figure 5:**
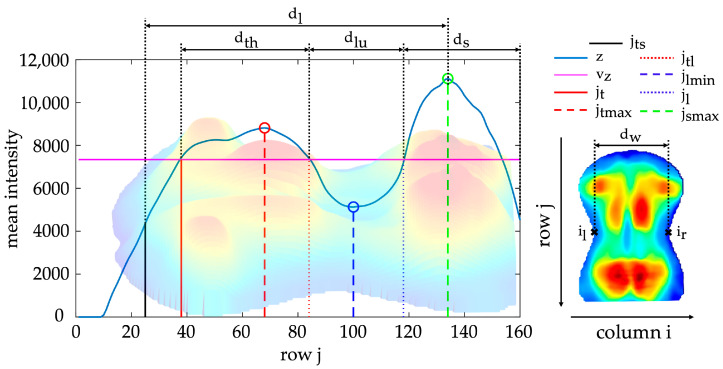
Mean intensity curve z(j) with marked landmarks (torso start $j_{\mathrm{ts}}$, thoracic regions start $j_{t}$, thoracic maximum $j_{\mathrm{tmax}}$, thoracolumbar transition $j_{\mathrm{tl}}$, lumbar minimum $j_{\mathrm{lmin}}$, lumbar regions end $j_{l}$, sacral maximum $j_{\mathrm{smax}}$, left reference position $i_{l}$, right reference position $i_{r}$) and reference distances (torso length $d_{l}$, torso width $d_{w}$, thoracic region length $d_{\mathrm{th}\;}$, lumbar region length $d_{\mathrm{lu}\;}$, sacral region length $d_{s\;}$); the displayed curve z(j) is based on the underlying three-dimensional back imprint.

The torso imprint was subdivided from cranial to caudal into anatomically associated regions according to Figure 5: thoracic region $d_{\mathrm{th}\;}$, lumbar region $d_{\mathrm{lu}}$ and sacral region $d_{s}$. The three regions were identified based on the mean intensity curve z(j) of the pressure intensities of all rows j within a range of $\Delta i_{z}=30\;\mathrm{px}$, according to Equation (6).

(6) $$z\left(j\right)=\frac{1}{{∆i}_{z}+1}\sum_{i=i_{\mathrm{sym}}-\frac{{\Delta i}_{z}}{2}}^{i_{\mathrm{sym}+\frac{{\Delta i}_{z}}{2}}}I(j,i)$$

The thoracic maximum $j_{\mathrm{tmax}}$, lumbar minimum $j_{\mathrm{lmin}}$ and sacral maximum $j_{\mathrm{smax}}$ were localised in the curve z(j) by extreme value analysis. The boundaries of the region were approximated using intersections with the mean value $v_{z}$ (Figure 5) according to Equation (7).

(7) $$v_{z}=\frac{1}{j_{\mathrm{lmin}}\;-\;j_{\mathrm{ts}}+1}\cdot \sum_{j=j_{\mathrm{ts}}}^{j_{\mathrm{lmin}}}z(j)$$

This marked the start of the thoracic region $j_{t}$, the thoracolumbar transition $j_{\mathrm{tl}}$ and the end of the lumbar region $j_{l}$. In addition, landmarks were defined as a reference position of the left $i_{l}$ and right torso $i_{r}$ boundaries. These reference points corresponded to the points with the maximum concave curvature along the contour of the ROI. The resulting landmarks were intended for localising of several morphological structures as well as determining reference distances along the longitudinal axis.

### 2.6. Parameter Extraction

Several diagnostic medical features exist for the identifying and assessing of postural disorders, such as scoliosis. These features commonly denote an asymmetry or imbalance of various structures of the torso. Table 1 lists commonly used features for this purpose and shows the association with the extracted structures in the torso imprint. For each diagnostic medical feature, different parameters and associated extraction approaches were proposed that could be utilised with torsobarography to assess postural disorders. The parameter extraction started with the segmentation of specific regions within the pressure image utilising a subject-specific, adaptive threshold value. Then, techniques of peak detection and edge detection were applied to identify the anatomically relevant structures within the selected regions. Structural shifts, angular relationships, gradients and pressure ratios characterised the shape and symmetry of the identified structures. The following section details the fundamental mathematical background underlying the extraction and parameterisation of these anatomical structures.

#### 2.6.1. Approximation of the Frontal and Sagittal Spine Shape

**Figure 6:**
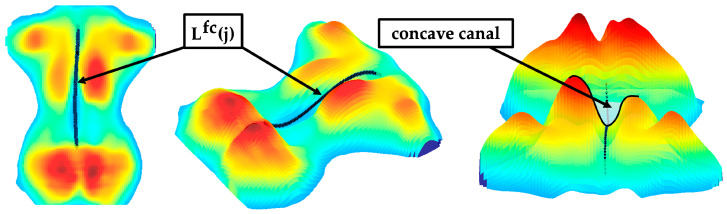
Sample of an approximated frontal curve of the spine $L^{\mathrm{fc}}\left(j\right)$ with highlighted canal.

The paraspinal muscles of the back form a concave canal in the region of the spinous processes when viewed in the frontal plane. An extreme value analysis localised the concave canal (Figure 6) to approximate the frontal curve of the spine. Only pressure intensities in the centred range $\Delta i_{z}$ around the symmetry axis $i_{\mathrm{sym}}$ within $I_{\mathrm{roi}}\left(j,i\right)$ were included. In general, a centrally located minimum existed in this range in every row between $j_{t}$ and $j_{l}$. Multiple extrema were sometimes be detected due to the variability of the dorsal anatomy and artifacts in the pressure image. The minimum closest to $i_{\mathrm{sym}}$ was selected as the centre of the concave canal. The detected points were smoothed via a fifth-degree polynomial regression, yielding the approximated frontal curve of the spine $L_{\mathrm{fc}}\left(j\right)$ (Figure 6).

A linear regression $L_{\mathrm{fc},s}\left(j\right)$ generalised the orientation (skew) of $L_{\mathrm{fc}}\left(j\right)$. Lateral deviations of the frontal curve $L_{\mathrm{fc}}\left(j\right)$ were quantified along as well as between the thoracic and lumbar region. The extracted parameters for assessing the approximate frontal curve of the spine were as follows:

- ${\mathrm{FC}}_{1}$: Summation of the absolute differences between adjacent points of $L_{\mathrm{fc}}\left(j\right)$
  (8) $${\mathrm{FC}}_{1\;}=\sum_{j_{t}}^{j_{l\;}}\left|L_{\mathrm{fc}}(j)\;-L_{\mathrm{fc}}\left(j+1\right)\right|$$
- ${\mathrm{FC}}_{2}$: Variance of $L_{\mathrm{fc}}\left(j\right)$
  (9) $${\mathrm{FC}}_{2}=\mathrm{var}(L_{\mathrm{fc}}(j))\;\mathrm{for}\;j\;\in [j_{t},j_{l}]$$
- ${\mathrm{FC}}_{3}$: Mean value of $L_{\mathrm{fc}}\left(j\right)$ in thoracic region in relation to mean value of $L_{\mathrm{fc}}\left(j\right)$ in lumbar region
  (10) $${\mathrm{FC}}_{3}=\frac{\frac{1}{d_{th}}\cdot \sum_{j_{t}}^{j_{\mathrm{tl}}}L_{\mathrm{fc}}\left(j\right)}{\frac{1}{d_{lu}}\cdot \sum_{j_{\mathrm{tl}}}^{j_{l}}L_{\mathrm{fc}}\left(j\right)}$$
- ${\mathrm{FC}}_{4}$: Lateral deviation of $L^{\mathrm{fc}}\left(j\right)$ in the upper thoracic and lower lumbar region
  (11) $${\mathrm{FC}}_{4}=\left|\frac{1}{10}\cdot \left(\sum_{j_{t}}^{j_{t}+9}L_{\mathrm{fc}}\left(j\right)\;-\sum_{j=j_{l}\;-9}^{j_{l}}L_{\mathrm{fc}}\left(j\right)\right)\right|$$
- ${\mathrm{FC}}_{5}$: Maximum deviation of $L^{\mathrm{fc}}\left(j\right)$ to the symmetry axis
  (12) $${\mathrm{FC}}_{5}=\underset{\forall j\in \;[j_{t},j_{l}]}{\max}\left|L_{\mathrm{fc}}\left(j\right)\;-i_{\mathrm{sym}}\right|$$
- ${\mathrm{FC}}_{6}$: Summation of the deviations of the points of $L_{\mathrm{fc}}\left(j\right)$ to the points of $L_{\mathrm{fc},s}\left(j\right)$
  (13) $${\mathrm{FC}}_{6}=\sum_{j_{t}}^{j_{l}}\left|L_{\mathrm{fc}}\left(j\right)\;-L_{\mathrm{fc},s}\left(j\right)\right|$$
- ${\mathrm{FC}}_{7}$: Absolute value of the first coefficient of $p_{\mathrm{fc},s}$ describing the slope of $L_{\mathrm{fc},s}\left(j\right)$ ($n_{\mathrm{fc},s}$ defines the vertical intersection of the following linear equation)
  (14) $$p_{\mathrm{fc},s}=\left|{\mathrm{FC}}_{7\;}\right|\cdot \;j+n_{\mathrm{fc},s}$$

Thoracic kyphosis and lumbar lordosis caused pressure intensities to be higher in the thoracic region than in the lumbar region. The resulting distribution of pressure intensities was morphologically similar to the sagittal shape of the spine (Figure 5). Therefore, the mean intensity curve z(j) between landmarks $j_{t}$ and $j_{l}$ was analysed as an approximation. To evaluate the curvature of z(j), linear regressions were performed for the intervals ${[j}_{t},j_{\mathrm{tmax}}]$, ${[j}_{\mathrm{tmax}},j_{\mathrm{tl}}]$, ${[j}_{\mathrm{tl}},j_{\mathrm{lmin}}]$ and ${[j}_{\mathrm{lmin}},j_{l}]$ (Figure 5). Angles were calculated from the resulting slopes to approximate the sagittal shape of the spine:

- ${\mathrm{SC}}_{1}$: Angle describing the shape of the thoracic curve (associated with the kyphosis angle) is determined by the slope of mean pressure intensities between ${[j}_{t},j_{\mathrm{tmax}}]$ as $m_{t1}$ and ${[j}_{\mathrm{tmax}},j_{\mathrm{tl}}]$ as $m_{t2}$
  (15) $${\mathrm{SC}}_{1}=\arctan\left|\frac{m_{t1\;}-m_{t2}}{1+m_{t1}\;\cdot \;m_{t2}}\right|$$
- ${\mathrm{SC}}_{2}$: Angle describing the shape of the lumbar curve (associated with the lordosis angle) is determined by the slope of mean pressure intensities between ${[j}_{\mathrm{tl}},j_{\mathrm{lmin}}]$ as $m_{l1}$ and ${[j}_{\mathrm{lmin}},j_{l}]$ as $m_{l2}$
  (16) $${\mathrm{SC}}_{2}=\arctan\left|\frac{m_{l1}-m_{l2}}{1+m_{l1}\;\cdot {\;m}_{l2}}\right|$$

The mean pressure intensities z(j) in the thoracic and lumbar regions were compared relative to each other to parameterise the imbalance between thoracic kyphosis and lumbar lordosis. Afterwards, the slope between the thoracic maximum and the lumbar minimum was quantified to describe the transition from thoracic kyphosis to lumbar lordosis.

- ${\mathrm{SC}}_{3}$: Ratio of thoracic maximum to lumbar minimum
  (17) $${\mathrm{SC}}_{3}=\frac{z\left(j_{\mathrm{tmax}}\right)}{z(j_{\mathrm{lmin}})}$$
- ${\mathrm{SC}}_{4}$: Ratio of summed thoracic pressure intensities to summed lumbar pressure intensities
  (18) $${\mathrm{SC}}_{4}=\frac{\sum_{j_{t}}^{j_{\mathrm{tl}}}z(j)\;}{\sum_{j_{\mathrm{tl}}}^{j_{l}}z(j)\;}$$
- ${\mathrm{SC}}_{5}$: Ratio of mean thoracic pressure intensities to mean lumbar pressure intensities
  (19) $${\mathrm{SC}}_{5}=\frac{\frac{1}{d_{th}}\cdot \sum_{j_{t}}^{j_{\mathrm{tl}}}z(j)\;}{\frac{1}{d_{lu}}\cdot \sum_{j_{\mathrm{tl}}}^{j_{l}}z(j)\;}$$
- ${\mathrm{SC}}_{6}$: Slope between thoracic maximum and lumbar minimum
  (20) $${\mathrm{SC}}_{6}=\frac{z\left(j_{\mathrm{tmax}}\right)\;-\;z(j_{\mathrm{lmin}})}{j_{\mathrm{tmax}}\;-j_{\mathrm{lmin}}}$$

Additionally, the extent of the thoracic maximum and lumbar minimum were separately quantified relative to the reference value $v_{z}$.

- ${\mathrm{SC}}_{7}$: Ratio of thoracic maximum to reference value $v_{z}$
  (21) $${\mathrm{SC}}_{7}=\frac{z(j_{\mathrm{tmax}})}{v_{z}}$$
- ${\mathrm{SC}}_{8}$: Ratio of lumbar minimum to reference value $v_{z}$
  (22) $${\mathrm{SC}}_{8}=\frac{z(j_{lmin})}{v_{z}}$$

#### 2.6.2. Morphological Structures of the Thoracic Region

**Figure 7:**
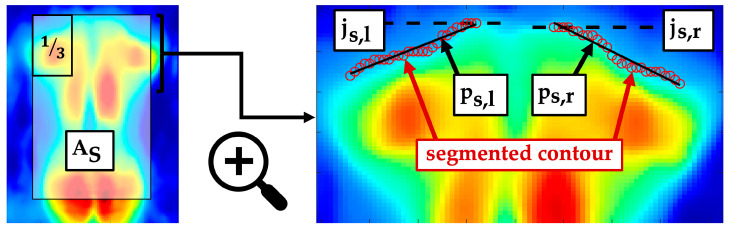
Segment $A_{S}$ and an enlarged section with marked shoulder contours, their linear regressions $p_{s,l/r}$ and the shoulder edges $j_{s,l/r}$.

The shoulders bulged ventrally from the lying surface due to protraction and thoracic kyphosis. The cranially located contour of the torso imprint was assumed to approximate the shoulder contour. The shoulder regions were located in the upper left and upper right third of the segment $A_{S}$, shown in Figure 7. The segment $A_{S}$ was defined by the intervals $\left[j_{\mathrm{ts}},j_{\mathrm{smax}}\right]$ and $[i_{l}\;-10,i_{r}+10]$. Both shoulder regions were adaptively segmented based on their mean value. The left upper shoulder edge $S_{0,l}$ as well as the right upper shoulder edge $S_{0,r}$ were identified as $j_{s,l}$ and $j_{s,r}$, respectively. Two independent linear regressions, $p_{s,l/r}$ with the slope coefficients $m_{s,l/r}$, generalised the morphology of the two shoulder contours. A difference in height and shape of the detected shoulder contours was quantified to identify potentially uneven shoulders.

- $S_{1}$: Vertical shift between left and right shoulder edges
  (23) $$S_{1}=\left|S_{0,l}-S_{0,r}\right|=\left|j_{s,l}-j_{s,r}\right|$$
- $S_{2}$: Maximum ratio of the slope coefficients of $p_{s,l/r}$
  (24) $$S_{2}=\max\left(\left|\frac{S_{2,l}}{S_{2,r}}\right|,\;\left|\frac{S_{2,r}}{S_{2,l}}\right|\right)=\max\left(\left|\frac{m_{s,l}}{m_{s,r}}\right|,\;\left|\frac{m_{s,r}}{m_{s,l}}\right|\right)$$

**Figure 8:**
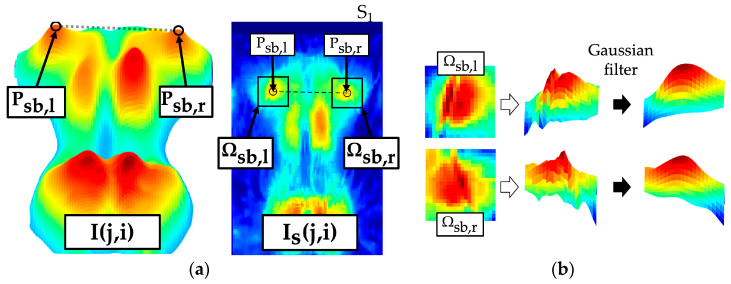
Localisation of scapula (left shoulder blade centre $P_{\mathrm{sb},l}(j_{\mathrm{sb},l},i_{\mathrm{sb},l})$, right shoulder blade centre $P_{\mathrm{sb},r}(j_{\mathrm{sb},r},i_{\mathrm{sb},r})$) and extraction of the scapula region (left scapula segment $\Omega_{\mathrm{sb},l}$, right scapula segment $\Omega_{\mathrm{sb},r}$) from the pressure image $I_{s}\left(j,i\right)$. (**a**) Marked shoulder blade segments; (**b**) effect of the Gaussian filter.

The scapulae caused accentuated maxima in the upper thoracic region. The scapula maxima were assessed within $I_{s}\left(j,i\right)$. Examination ranges were defined in the interval ${[j}_{t},j_{\mathrm{tl}}]$ depending on the symmetry axis $i_{\mathrm{sym}}$ for left $[1,i_{\mathrm{sym}}\;-\frac{i_{r}-i_{l}}{4}]$ and right scapula interval $[i_{\mathrm{sym}}+\frac{i_{r}\;-i_{l}}{4},100]$. Thereby, pressure maxima caused by the ribs and peripheral back muscles were excluded. Both shoulder blade regions were roughly presegmented with the threshold value (tv). The scapula was represented by a prominent region with several convex and concave contours. A Gaussian filter (3 × 3, σ=2) smoothed the region (Figure 8a) so that only the most prominent maxima were highlighted for each shoulder blade. An extreme value analysis identified the two highest maxima ($P_{\mathrm{sb},\max_{1}}$, $P_{\mathrm{sb},\max_{2}})$ for each region. The maximum selected as the shoulder blade centre $P_{\mathrm{sb},l/r}(j_{\mathrm{sb},l/r},i_{\mathrm{sb},l/r})$ was the one located more laterally away from the axis of symmetry $i_{\mathrm{sym}}$ and had a higher pressure intensity.

(25) $$\left({\mathrm{SB}}_{0,\mathrm{vl}},{\;\mathrm{SB}}_{0,\mathrm{hl}})=(j_{\mathrm{sb},l},\;\left|i_{\mathrm{sb},l}-i_{\mathrm{sym}}\right|\right)$$

(26) $$\left({\mathrm{SB}}_{0,\mathrm{vr}},{\;\mathrm{SB}}_{0,\mathrm{vr}})=(j_{\mathrm{sb},r},\;\left|i_{\mathrm{sb},r}-i_{\mathrm{sym}}\right|\right)$$

For the parameterisation, a symmetrical segment $\Omega_{\mathrm{sb},l/r}$ of 11 × 11 pixels was defined at each of the localised shoulder blade centres (Figure 8b) within $I_{s}\left(j,i\right)$. The following parameters quantified the symmetry of the scapulae with respect to position and intensity. The greater the parameters, the more asymmetrical the structures that are detected. This in turn indicates unequal shoulders or unequal protrusion.

- ${\mathrm{SB}}_{1}$: Vertical shift of the localised scapula centres
  (27) $${\mathrm{SB}}_{1}=\left|{\mathrm{SB}}_{0,\mathrm{vl}}-\;{\mathrm{SB}}_{0,\mathrm{vr}}\right|=\left|j_{\mathrm{sb},l}\;-j_{\mathrm{sb},r}\right|$$
- ${\mathrm{SB}}_{2}$: Horizontal shift of the localised scapula centres to the axis of symmetry
  (28) $${\mathrm{SB}}_{2}=\left|{\mathrm{SB}}_{0,\mathrm{hl}}\;-\;{\mathrm{SB}}_{0,\mathrm{hr}}\right|=\left|\left(i_{\mathrm{sb},r}-\;i_{\mathrm{sym}}\right)\;-\left(i_{\mathrm{sym}}\;-\;i_{\mathrm{sb},l}\right)\right|$$
- ${\mathrm{SB}}_{3}$: Angle between the localised scapula centres
  (29) $${\mathrm{SB}}_{3}=\arctan\left|\frac{j_{\mathrm{sb},r}\;-j_{\mathrm{sb},l}}{i_{\mathrm{sb},r}\;-\;i_{\mathrm{sb},l}}\right|$$
- ${\mathrm{SB}}_{4}$: Maximum ratio of scapula maxima between $\Omega_{\mathrm{sb},l}$ and $\Omega_{\mathrm{sb},r}$
  (30) $${\mathrm{SB}}_{4}=\max\left(\frac{{\mathrm{SB}}_{4,l}}{{\mathrm{SB}}_{4,r}},\;\frac{{\mathrm{SB}}_{4,r}}{{\mathrm{SB}}_{4,l}}\right)=\max\left(\frac{\underset{j,i\in \;\Omega_{\mathrm{sb},l}}{\max}I_{s}\left(j,i\right)}{\underset{j,i\in \;\Omega_{\mathrm{sb},r}}{\max}I_{s}\left(j,i\right)},\frac{\underset{j,i\in \;\Omega_{\mathrm{sb},r}}{\max}I_{s}\left(j,i\right)}{\underset{j,i\in \;\Omega_{\mathrm{sb},l}}{\max}I_{s}\left(j,i\right)}\right)$$
- ${\mathrm{SB}}_{5}$: Maximum ratio of mean pressure intensities between $\Omega_{\mathrm{sb},l}$ and $\Omega_{\mathrm{sb},r}$
  (31) $${\mathrm{SB}}_{5}=\max\left(\frac{{\mathrm{SB}}_{5,l}}{{\mathrm{SB}}_{5,r}},\;\frac{{\mathrm{SB}}_{5,r}}{{\mathrm{SB}}_{5,l}}\right)=\max\left(\frac{\frac{1}{\left|\Omega_{\mathrm{sb},l}\right|}\cdot \sum_{j,i\in \;\Omega_{\mathrm{sb},l}}I_{s}\left(j,i\right)}{\frac{1}{\left|\Omega_{\mathrm{sb},r}\right|}\cdot \sum_{j,i\in \;\Omega_{\mathrm{sb},r}}I_{s}\left(j,i\right)},\frac{\frac{1}{\left|\Omega_{\mathrm{sb},r}\right|}\cdot \sum_{j,i\in \;\Omega_{\mathrm{sb},r}}I_{s}\left(j,i\right)}{\frac{1}{\left|\Omega_{\mathrm{sb},l}\right|}\cdot \sum_{j,i\in \;\Omega_{\mathrm{sb},l}}I_{s}\left(j,i\right)}\right)$$

**Figure 9:**
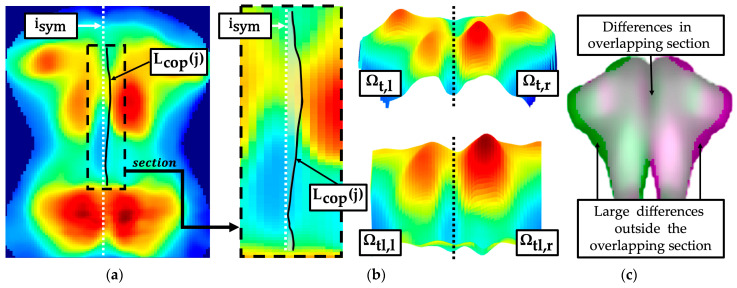
Assessment of torso symmetry in the pressure image with centre of pressure curve $L_{\mathrm{cop}}\left(j\right)$ and symmetry comparison of the two sides of the body (left thoracic segment $\Omega_{t,l}$, right thoracic segment $\Omega_{t,r}$, left combined thoracolumbar segment $\Omega_{\mathrm{tl},l}$, right combined thoracolumbar segment $\Omega_{\mathrm{tl}}$). (**a**) Centre of pressure curve; (**b**) left and right segments; (**c**) mirror symmetry.

The symmetry difference of the pressure intensity distribution was analysed along the longitudinal axis for major segments of the torso imprint (Figure 9b): thoracic segment $\Omega_{t}$, lumbar segment $\Omega_{l}$, combined segment $\Omega_{\mathrm{tl}}$ from $\Omega_{t}$ and $\Omega_{l}$ caudal to scapula section $\Omega_{\mathrm{sb},l/r}$. A centre of pressure curve $L_{\mathrm{cop}}\left(j\right)$ (Figure 9a) was determined for the segments $\Omega_{t}$ and $\Omega_{l}$ within the interval of the torso width ${[i}_{l},i_{r}]$. The segment $\Omega_{t}$ as well as the segment $\Omega_{\mathrm{tl}}$ were divided into left and right segments (Figure 9b), according to Equations (32)–(35).

(32) $$\Omega_{t,l}=\left\{\left(j,i\right)|j_{\mathrm{ts}}\leq j\leq j_{\mathrm{lmin}},\;i_{l}\;-\;10\leq i<i_{\mathrm{sym}}\right\}\cap I_{\mathrm{roi}}\left(j,i\right)$$

(33) $$\Omega_{t,r}=\left\{\left(j,i\right)|j_{\mathrm{ts}}\leq j\leq j_{\mathrm{lmin}},\;i_{\mathrm{sym}}<i\leq i_{r}+10\;\right\}\cap I_{\mathrm{roi}}\left(j,i\right)$$

(34) $$\Omega_{\mathrm{tl},l}=\left\{\left(j,i\right)|j_{\mathrm{sb},l}+10\leq j\leq j_{l},\;i_{\mathrm{sym}}\;-\;\frac{i_{r}\;-i_{l}}{2}\leq i<\;i_{\mathrm{sym}}\right\}\cap I_{\mathrm{roi}}\left(j,i\right)$$

(35) $$\Omega_{\mathrm{tl},r}=\left\{\left(j,i\right)|j_{\mathrm{sb},r}+10\leq j\leq j_{l},\;i_{\mathrm{sym}}<i\leq \;i_{\mathrm{sym}}+\frac{i_{r}\;-\;i_{l}}{2}\right\}\cap I_{\mathrm{roi}}\left(j,i\right)$$

An asymmetric torso imprint, such as one that may occur with a rib hump, was characterised by an unequal distribution of surface area and intensity in relation to the symmetry axis. The inequality was quantified using the following parameters:

- ${\mathrm{TS}}_{1}$: Summed deviation between centre of pressure curve $L_{\mathrm{cop}}\left(j\right)$ and symmetry axis
  (36) $${\mathrm{TS}}_{1}=\sum_{j_{k}}^{j_{l}}\left(L_{\mathrm{cop}}\left(j\right)-i_{\mathrm{sym}}\right)$$
- ${\mathrm{TS}}_{2}$: Maximum ratio between the mean values of $\Omega_{\mathrm{tl},l}$ and $\Omega_{\mathrm{tl},r}$
  (37) $${\mathrm{TS}}_{2}=\max\left(\frac{{\mathrm{TS}}_{2,l}}{{\mathrm{TS}}_{2,r}},\;\frac{{\mathrm{TS}}_{2,r}}{{\mathrm{TS}}_{2,l}}\right)=\max\left(\frac{\frac{1}{\left|\Omega_{\mathrm{tl},l}\right|}\cdot \sum_{j,i\in \;\Omega_{\mathrm{tl},l}}I_{\mathrm{roi}}\left(j,i\right)}{\frac{1}{\left|\Omega_{\mathrm{tl},r}\right|}\cdot \sum_{j,i\in \;\Omega_{\mathrm{tl},r}}I_{\mathrm{roi}}\left(j,i\right)},\frac{\frac{1}{\left|\Omega_{\mathrm{tl},r}\right|}\cdot \sum_{j,i\in \;\Omega_{\mathrm{tl},r}}I_{\mathrm{roi}}\left(j,i\right)}{\frac{1}{\left|\Omega_{\mathrm{tl},l}\right|}\cdot \sum_{j,i\in \Omega_{\mathrm{tl},l}}I_{\mathrm{roi}}\left(j,i\right)}\right)$$
- ${\mathrm{TS}}_{3}$: Maximum ratio between maximum pressure intensities of $\Omega_{\mathrm{tl},l}$ and $\Omega_{\mathrm{tl},r}$
  (38) $${\mathrm{TS}}_{3}=\max\left(\frac{{\mathrm{TS}}_{3,l}}{{\mathrm{TS}}_{3,r}},\;\frac{{\mathrm{TS}}_{3,r}}{{\mathrm{TS}}_{3,l}}\right)=\max\left(\frac{\underset{j,i\in \;\Omega_{\mathrm{tl},l}}{\max}I_{\mathrm{roi}}\left(j,i\right)}{\underset{j,i\in \Omega_{\mathrm{tl},r}}{\max}I_{\mathrm{roi}}\left(j,i\right)},\frac{\underset{j,i\in \;\Omega_{\mathrm{tl},r}}{\max}I_{\mathrm{roi}}\left(j,i\right)}{\underset{j,i\in \Omega_{\mathrm{tl},l}}{\max}I_{\mathrm{roi}}\left(j,i\right)}\right)$$
- ${\mathrm{TS}}_{4}$: Maximum ratio between the summed pressure intensities of $\Omega_{t,l}$ and $\Omega_{t,r}$
  (39) $${\mathrm{TS}}_{4}=\max\left(\frac{{\mathrm{TS}}_{4,l}}{{\mathrm{TS}}_{4,r}},\;\frac{{\mathrm{TS}}_{4,r}}{{\mathrm{TS}}_{4,l}}\right)=\max\left(\frac{\sum_{j,i\in \;\Omega_{t,l}}I_{\mathrm{roi}}\left(j,i\right)}{\sum_{j,i\in \;\Omega_{t,r}}I_{\mathrm{roi}}\left(j,i\right)},\frac{\sum_{j,i\in \;\Omega_{t,r}}I_{\mathrm{roi}}\left(j,i\right)}{\sum_{j,i\in \Omega_{t,l}}I_{\mathrm{roi}}\left(j,i\right)}\right)$$

An approach was implemented that quantified the difference between the torso imprint $I_{\mathrm{roi}*}\left(j,i\right)$ segmented by the mean pressure intensity and the corresponding torso imprint $I_{\mathrm{roi}*}^{*}\left(j,i\right)$ mirrored to the symmetry axis. Differences were exclusively quantified in the interval $\left[j_{\mathrm{ts}},j_{\mathrm{lmin}}\right]$ within the overlapping sections (Figure 9c).

- ${\mathrm{TS}}_{5}$: Summation of the percentage differences between $I_{\mathrm{roi}*}\left(j,i\right)$ and $I_{\mathrm{roi}*}^{*}\left(j,i\right)$ normalised to power of the set $\Omega_{\mathrm{roi}*}$ within the overlapping section
  (40) $${\mathrm{TS}}_{5}=\frac{1}{\left|\Omega_{\mathrm{roi}*}\right|}\;\cdot \;\sum_{\forall i,j\;\in [j_{\mathrm{ts}},j_{\mathrm{lmin}}]}\left|1\;-\frac{I_{\mathrm{roi}*}\left(j,i\right)}{I_{\mathrm{roi}*}^{*}\left(j,i\right)}\right|\mathrm{with}{\;\Omega }_{\mathrm{roi}*}=\left\{I_{\mathrm{roi}*}|I_{\mathrm{roi}*\;}>\;0\right\}$$

#### 2.6.3. Morphological Structures of the Lumbar Region

**Figure 10:**
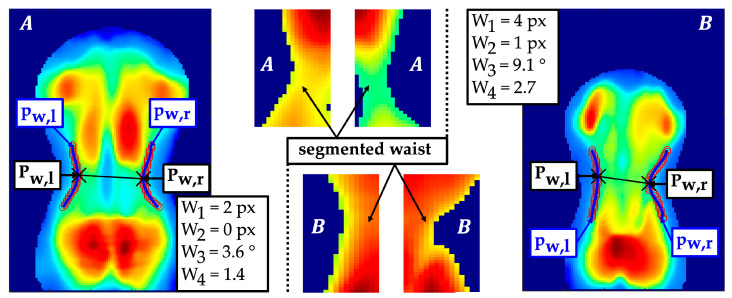
Segmentation of the waist contours with polynomial regressions $p_{w,l/r}$, localisation of waist contour centres $P_{w,l/r}(j_{w,l/r},i_{w,l/r})$ and example of a rather symmetrical waist contour (**A**) and an asymmetrical waist contour (**B**).

Anatomically, there is a concave curvature from lateral to medial between the thorax and the hip in the region of the waist. The extraction of the waist contour was performed using an adaptive threshold-based segmentation (Figure 10). The threshold values were calculated based on the mean pressure intensities of the left and right waist imprints within $I_{\mathrm{roi}}\left(j,i\right)$. Two fourth-degree polynomial regressions $p_{w,l/r}$ approximated the curve of the waist contour. The minima within the lumbar region of $p_{w,l}$ and $p_{w,r}$ were detected, which served as centres $P_{w,l/r}(j_{w,l/r},i_{w,l/r})$ of the waist contours.

(41) $$\left(W_{0,\mathrm{vl}},{\;W}_{0,\mathrm{hl}})=(j_{w,l},\;\left|i_{w,l}-i_{\mathrm{sym}}\right|\right)$$

(42) $$\left(W_{0,vr},{\;W}_{0,\mathrm{hr}})=(j_{w,r},\;\left|i_{w,r}-i_{\mathrm{sym}}\right|\right)$$

For a robust shape comparison of the two waist contours, an additional second-degree polynomial regression $p_{\mathrm{ws},l/r}$ was performed, which produced the quadratic coefficients $a_{\mathrm{ws},l/r}$. The position of the waist centres and waist shape were quantified with respect to their symmetry properties:

- $W_{1}$: Vertical shift of the waist centres
  (43) $$W_{1}=\left|W_{0,\mathrm{vl}\;}-W_{0,\mathrm{vr}}\right|=\left|j_{w,l}\;-\;j_{w,r}\right|$$
- $W_{2}$: Difference horizontal shift of the waist centres to the symmetry axis
  (44) $$W_{2}=\left|W_{0,\mathrm{hl}}-\;W_{0,\mathrm{hr}}\right|=\left|\left(i_{w,r}-i_{\mathrm{sym}}\right)-\left(i_{\mathrm{sym}}\;-i_{w,l}\right)\right|$$
- $W_{3}$: Angle between both waist centres
  (45) $$W_{3}=\arctan\left|\frac{j_{w,l}\;-\;j_{w,r}}{i_{w,l}\;-\;i_{w,r}}\right|$$
- $W_{4}$: Maximum ratio of the quadratic coefficients of the second-degree polynomial regression of the left and right waist contour
  (46) $$W_{4}=\max\left(\left|\frac{W_{4,l}}{W_{4,r}}\right|,\;\left|\frac{W_{4,r}}{W_{4,l}}\right|\right)=\max\left(\left|\frac{a_{\mathrm{ws},l}}{a_{\mathrm{ws},r}}\right|,\;\left|\frac{a_{\mathrm{ws},r}}{a_{\mathrm{ws},l}}\right|\right)$$

#### 2.6.4. Morphological Structures of the Sacral Region

**Figure 11:**
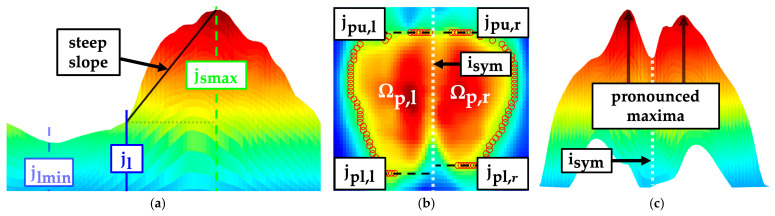
Sagittal (lumbar minimum $j_{\mathrm{lmin}}$, lumbar regions end $j_{l}$, sacral maximum $j_{\mathrm{smax}}$), frontal (left sacral region $\Omega_{p,l}$, right sacral region $\Omega_{p,r}$, upper edges of sacral imprint $j_{\mathrm{pu},l/r}$, lower edges of sacral imprint $j_{\mathrm{pl},l/r}$) and transversal view of the pelvic imprint. (**a**) Sagittal slope in the sacral region; (**b**) upper and lower edges; (**c**) pressure symmetry.

The flexion of the knees during the measurement procedure results in the majority of the weight being supported by the sacrum. A secondary pressure zone emerges beneath the gluteal muscle. Both pressure zones overlap in the sacral region. A steep slope of pressure intensities was noted from the transition of the lumbar region to the sacral region (Figure 11a). This slope may be indicative of the pelvic alignment in the sagittal plane. The slope was quantified by the linear regression $p_{\mathrm{pt}}$ of the mean intensity curve z(j) in the interval ${[j}_{l},j_{\mathrm{smax}}]$. The pressure imprint was adaptively segmented based on the mean value of the pressure intensities in the sacral region. Subsequently, it was divided into left $\Omega_{p,l}$ and right sacral region $\Omega_{p,r}$ depending on the symmetry axis $i_{\mathrm{sym}}$ (Figure 11b). Upper edges $j_{\mathrm{pu},l/r}$ and lower edges $j_{\mathrm{pl},l/r}$ of both sacral imprints were localised to identify a frontal shift between both body sides.

(47) $$\left(P_{0,\mathrm{ul}},{\;P}_{0,\mathrm{ll}})=(j_{pu,l},\;j_{pl,l}\right)$$

(48) $$\left(P_{0,\mathrm{ur}},{\;P}_{0,\mathrm{lr}})=(j_{pu,r},\;j_{pl,r}\right)$$

The gluteal muscles typically caused a pronounced maximum in the left and right sacral region. The symmetry of the pressure intensities between the two regions was compared relative to each other to quantify an asymmetric pressure distribution (Figure 11c). This could possibly be caused by a structurally induced rotation or torsion of the pelvis.

- $P_{1}$: Slope of $p_{\mathrm{pt}}$ in the interval ${[j}_{l},j_{\mathrm{smax}}]$
  (49) $$p_{\mathrm{pt}}=P_{1}\cdot j+n_{\mathrm{pt}}$$
- $P_{2}$: Summed vertical shift of the upper and lower edges of the segmented sacral imprint
  (50) $$P_{2}=\left|P_{0,\mathrm{ul}}\;-\;P_{0,\mathrm{ur}}\right|+\left|P_{0,\mathrm{ll}}\;-{\;P}_{0,\mathrm{lr}}\right|=\left|j_{\mathrm{pu},l}\;-\;j_{\mathrm{pu},r}\right|+\left|j_{\mathrm{pl},l}\;-\;j_{\mathrm{pl},r}\right|$$
- $P_{3}$: Maximum ratio between the summed pressure intensities of the left $\Omega_{p,l}$ and right sacral region $\Omega_{p,r}$
  (51) $$P_{3}=\max\left(\frac{P_{3,l}}{P_{3,r}},\;\frac{P_{3,r}}{P_{3,l}}\right)=\max\left(\frac{\sum_{i,j\epsilon \Omega_{p,l}}I_{\mathrm{roi}}\left(j,i\right)}{\sum_{i,j\epsilon \Omega_{p,r}}I_{\mathrm{roi}}\left(j,i\right)},\frac{\sum_{i,j\epsilon \Omega_{p,r}}I_{\mathrm{roi}}\left(j,i\right)}{\sum_{i,j\epsilon \Omega_{p,l}}I_{\mathrm{roi}}\left(j,i\right)}\right)$$
- $P_{4}$: Maximum ratio between the mean pressure intensities of the left $\Omega_{p,l}$ and right sacral region $\Omega_{p,r}$
  (52) $$P_{4}=\max\left(\frac{P_{4,l}}{P_{4,r}},\;\frac{P_{4,r}}{P_{4,l}}\right)=\max\left(\frac{\frac{1}{\left|\Omega_{p,l}\right|}\cdot \sum_{i,j\epsilon \Omega_{p,l}}I_{\mathrm{roi}}\left(j,i\right)}{\frac{1}{\left|\Omega_{p,r}\right|}\cdot \sum_{i,j\epsilon \Omega_{p,r}}I_{\mathrm{roi}}\left(j,i\right)},\frac{\frac{1}{\left|\Omega_{p,r}\right|}\cdot \sum_{i,j\epsilon \Omega_{p,r}}I_{\mathrm{roi}}\left(j,i\right)}{\frac{1}{\left|\Omega_{p,l}\right|}\cdot \sum_{i,j\epsilon \Omega_{p,l}}I_{\mathrm{roi}}\left(j,i\right)}\right)$$

### 2.7. Statistical Analysis

The reliability between the identified landmarks, resulting reference distances and localised morphological structures as well as their degrees and derived parameters was evaluated using the intraclass correlation coefficient ICC(1,1). The ICC was implemented according to [31]. The interpretation of ICC was based on the following criteria: excellent reliability (ICC ≥ 0.9), good reliability (0.75 ≤ ICC < 0.9), moderate reliability (0.5 ≤ ICC < 0.75) or poor reliability (ICC < 0.5) [32]. In addition, the confidence interval (95% CI) of ICC and the mean coefficient of variation (CV) were calculated. The consistency of the measures across the measurement repetitions was ranked by CV as follows: excellent consistency (CV ≤ 10%), good consistency (10% < CV ≤ 20%), moderate consistency (20% < CV ≤ 30%) or poor consistency (CV > 30%) [33]. Furthermore, length measurements and localised structures were assessed using the mean standard deviation (SD). The rating of SD was dependent on the resolution (1 px = 5.1 mm × 5.1 mm) of the sensor array: very small deviation (SD ≤ 1 px), small deviation (1 px < SD ≤ 2 px), moderate deviation (2 px < SD ≤ 3 px) or large deviation (SD > 3 px).

## 3. Results

### 3.1. Landmarks and Reference Distances

According to the mean CV, all extracted landmarks and reference distances showed a relative scatter of less than 10% (Table 2). In eight of nine cases, a good to excellent reliability level was achieved for the landmarks used to divide the torso imprint into different anatomically associated regions. On the longitudinal axis, the start of the thoracic region and the thoracic maximum could be extracted with excellent reliability. The mean SD revealed small deviations for both thoracic landmarks. The lumbar minimum and sacral maximum only achieved moderate reliabilities, resulting in a scattering of parameters based on these two landmarks. In particular, the lumbar minimum exhibited a comparatively large deviation, exceeding 3 px. In contrast, the torso width could be detected with excellent reliability. Accordingly, the ICC values for the reference positions included in the calculation were high as well. Left and right reference positions had an SD less than 1 px and could therefore be identified with high precision considering the resolution of the sensor mat. The reference distances of all three regions exhibited a similarly high reliability. The corresponding SD values were classified as a low to moderate deviation.

### 3.2. Approximated Frontal and Sagittal Spine Curve

The variance in the identified curve (${\mathrm{FC}}_{2}$) was the only parameter with a moderate reliability for the approximated frontal curve of the spine. The other parameters achieved poor reliability (Table 3). The grand mean of the ratio between the thoracic and lumbar frontal curves (${\mathrm{FC}}_{3}$) indicated that the thoracic curve equals the lumbar curve (${\mathrm{FC}}_{3}$ = 1). In addition, the relative scatter for ${\mathrm{FC}}_{3}$ was very low at approximately 1.3%. The summation of the absolute differences of the frontal curve (${\mathrm{FC}}_{1}$) achieved poor reliability and consistency according to ICC and CV but bordered on a moderate reliability level. Conversely, nearly all parameters describing the sagittal curve of the spine exhibited an ICC above 0.800, indicating a high level of reliability. The associated kyphosis angle (${\mathrm{SC}}_{1}$) was much more reliable (ICC = 0.855) than the associated lordosis angle (${\mathrm{SC}}_{2}$). ${\mathrm{SC}}_{2}$ was, furthermore, the only parameter of the sagittal curve with just a moderate level of reliability (ICC = 0.650). The parameters associated with sagittal imbalance (${\mathrm{SC}}_{3}$–${\mathrm{SC}}_{5}$) along with degrees of thoracic (${\mathrm{SC}}_{6}$) and lumbar pressure (${\mathrm{SC}}_{7}$) not only achieved high ICC values but also displayed a low CV, indicating consistency from good to excellent.

### 3.3. Thoracic Region

Positions of the left ($S_{0,l}$) and right ($S_{0,r}$) shoulder contour were reliably extracted with an ICC of 0.87 (Table 4). The relative scatter of the two upper shoulder edges was low with a CV of less than 7%, but the deviation was moderate. Although a small SD of about 0.8 px was determined for $S_{1}$, the grand mean of the vertical deviation of 1.3 px was within the SD of the localised upper shoulder edges. A similar pattern was noted for the parameters quantifying the positional shift of the scapulae (${\mathrm{SB}}_{0,\mathrm{vl}/r}$). Although the vertical positions of the left and right scapulae were reliably detected with an ICC of 0.87, the SDs of the vertical positions were larger than the grand mean of the vertical shift. It was also notable that the horizontal positions of the scapulae were detected with slightly less reliability than the vertical positions. Nevertheless, the horizontal scapula positions were assigned to an upper moderate (${\mathrm{SB}}_{0,\mathrm{hr}}$) to good (${\mathrm{SB}}_{0,\mathrm{hl}}$) reliability level. The grand mean angle between the two scapular centres was approximately 2.9°, indicating minimal asymmetry in the localised positions among the group of participants.

A poor level of reliability was obtained for the parameters of the shoulder contour ($S_{2}$) as well as for the symmetry comparison of the intensities of both scapular segments (${\mathrm{SB}}_{4}$, ${\mathrm{SB}}_{5}$). Only the ratio of the scapular maxima (${\mathrm{SB}}_{4}$) approached a moderate reliability with an ICC of 0.478. However, if the respective shoulder contour coefficient ($S_{2,l/r}$) or the pressure intensity of a scapula (${\mathrm{SB}}_{4,l/r}$, ${\mathrm{SB}}_{5,l/r}$) were evaluated for the left and right body side independently, moderate to good reliability levels were reached. Particularly for the scapula maxima ${\mathrm{SB}}_{4,l/r}$ and the mean intensities ${\mathrm{SB}}_{5,l/r}$, ICC values exceeded 0.800 and the CV was very low, peaking at only 3.4%, indicative of excellent consistency.

Four out of the five parameters used to quantify a generalised asymmetric pressure distribution across the torso achieved a moderate reliability. Only the centre of pressure deviation (${\mathrm{TS}}_{1}$) and the total percentage difference from the mirrored pressure image (${\mathrm{TS}}_{5}$) showed moderate consistencies between 20% and 30%. The CV of the remaining parameters of the torso symmetry indicated poor consistency. The grand mean value of ${\mathrm{TS}}_{5}$ revealed that the pressure intensities vary by an average of 11.5%, with a maximum standard deviation of 2.4%. When comparing the ICC values between the pressure intensities used to calculate ${\mathrm{TS}}_{2}$, ${\mathrm{TS}}_{3}$ and ${\mathrm{TS}}_{4}$, it was evident that the maximum (${\mathrm{TS}}_{2,l/r}$) and mean pressure intensities (${\mathrm{TS}}_{3,l/r}$) exhibit a higher ICC than the intensities summed over a larger segment like ${\mathrm{TS}}_{4,l/r}$. The ICC values for ${\mathrm{TS}}_{2,l/r}$ and ${\mathrm{TS}}_{3,l/r}$ indicated good to excellent reliability levels.

### 3.4. Lumbar Region

The horizontal coordinates of the waist centres ($W_{0,hl/r}$) were extracted with a higher reliability than the vertical coordinates ($W_{0,\mathrm{vl}/r}$). $W_{0,hl/r}$ showed good reliability with an ICC of at least 0.864, whereas $W_{0,\mathrm{vl}/r}$ exhibited moderate reliability (Table 5), with an ICC of 0.664. $W_{0,hl/r}$ were determined with a smaller SD of about 0.8 px than $W_{0,\mathrm{vl}/r}$ with an SD of 2.9 px. Relative scatter up to a maximum of 4.8% of the side-related coordinates suggested a consistent localisation of the waist centres. Nevertheless, the shifts $W_{1/2}$ and resulting angle $W_{3}$ between the two waist centres were quantified with poor reliability. However, the comparison parameter for the left and right waist contour indicated a moderate reliability with an ICC of 0.689. Likewise, the corresponding quadratic coefficients $W_{4,l/r}$ were extracted with similar reliability, with an ICC of at least 0.726.

### 3.5. Sacral Region

Upper edges $P_{0,\mathrm{ul}/r}$ and lower edges $P_{0,\mathrm{ll}/r}$ of the sacral imprint were extracted with moderate reliability (Table 6). The ICC of the upper edges was close to the next highest reliability level with up to 0.747. Moreover, the CV for all coordinates to bound the sacral imprint was quite low, with a maximum of 2%. Nevertheless, the vertical shift $P_{2}$ was not reliably identified. The slope of the pressure values of the sacral region $P_{1}$ was based on the same extracted structure as the parameters of the approximated sagittal spine curve and achieved a similarly good reliability with an ICC of 0.771. When examining the maximum ratios for the symmetry evaluation ($P_{3}$, $P_{4}$), it was noticeable that only very low ICC values were attained. Nonetheless, the side-dependent pressure intensities used for calculation attained a moderate ($P_{3,l/r}$) to good reliability level ($P_{4,l/r}$). For the mean left and mean right sacral pressure intensities ($P_{4,l/r}$), ICC values above 0.850 were reached. However, the grand mean of the ratio $P_{4}$ in combination with the side-dependent intensities indicated a very symmetrical distribution of the intensities in the sacral region.

## 4. Discussion

This study presented a novel approach to extract parameters associated with asymmetries of anatomical structures of the back. The aim was to evaluate the intra-observer reliability of these parameters as groundwork for the evaluation of torsobarography as a potential screening and diagnostic method. The results showed that the localisation of structures exhibits good to excellent reliability in terms of reproducibility. In most cases, standard deviations of the positions of a few pixels were attained (1 px to 2 px), indicating an accuracy in the order of millimetres. The characteristics as well as the contours of the dorsal torso imprint were mainly identified with a good reliability level and with a good consistency with respect to the relative scatter. However, specific asymmetries are challenging to identify within a subject group with predominantly no diagnosed postural deformities, resulting in a reliability level that was rather poor to moderate. Future studies will have to demonstrate whether the high reliability in the characterising and localising of anatomically associated structures in pressure images is sufficient to detect postural deformities and assess progression.

### 4.1. Intra-Observer Reliability of Different Parameter Groups

The challenging task of identifying asymmetries on the back is performed either by manual examination, X-ray or topographic methods such as rasterstereography. Similar to the rasterstereographic system Formetric 4D, postural reference points are extracted along the associated sagittal profile in torsobarography. The localised points share a common causal background; for instance, the location of the thoracic maximum of torsobarography is expected to correspond with the reference position of the kyphosis apex of Formetric 4D. Therefore, to contextualise the results, it is advisable to compare them with Formetric 4D. Upon comparison, it was evident that some of the landmarks investigated here, just like the Formetric 4D reference positions [21], had an excellent level of reliability or approached it. For parameters associated with trunk length, an ICC of 0.950 was reported for the commercial systems [21,22]. The ICC determined here was 0.800. The discrepancy is probably due to the reason that landmarks utilised for the calculation, such as the sacral maximum, had a comparatively low ICC of 0.680 compared to the other landmarks. Therefore, the beginning of the thoracic region (ICC = 0.917) and the end of the lumbar region (ICC = 0.855) are probably more appropriate landmarks for calculating torso length. Nonetheless, the other reference distances achieved good and excellent reliability levels. Therefore, it can be concluded that the anatomically associated landmarks and derived reference distances of torsobarography can be reproducibly extracted for an individual subject.

All parameters for assessing the frontal shape of the spine aim at quantifying the asymmetry of the identified curve. The corresponding ICC values, with a maximum of 0.57, indicated that this was only possible with a moderate level of reliability for a group of subjects who predominantly do not have any postural deformities. Under conditions of constant measurement precision, an increased ICC is expected to be observed when measuring a subject group with a broader variability in the considered parameter. In comparison, Tabard-Fougère et al. only achieved a moderate level of reliability (ICC = 0.700) for the scoliosis angle within Formetric 4D measurements, despite a sample group of 35 AIS patients [19]. Similarly, the study by Degenhardt et al., based on 30 adults without postural deformities, reported a relatively low ICC of 0.690 for the parameter of coronal imbalance [21]. Therefore, while expanding the dataset to include more scoliotic subjects may lead to higher ICC values, creating a dependable reconstruction of the frontal spine without X-ray or ultrasound-based systems (e.g., Scolioscan [34]) remains challenging.

Almost all parameters of the sagittal curve achieved good reliability. Therefore, they were more reliable than similar curvature parameters of the frontal plane. This was expected, as the sagittal curvature generally exhibits greater variability than the frontal curvature in a healthy population [35]. The evaluation of the reliability of ${\mathrm{SC}}_{1}$ and ${\mathrm{SC}}_{2}$ requires a comparison with literature-reported values for assessing kyphosis and lordosis angles. However, a direct interpretation as kyphosis and lordosis angles is precluded because of dissimilar scaling of the surface and pressure profiles in relation to the length of the spine. Nevertheless, it is likely that these parameters will demonstrate a robust correlation, given their shared calculation approach. The associated kyphosis angle ${\mathrm{SC}}_{1}$ calculated in this study exhibited a high level of reliability, with an ICC value approaching the threshold of excellent reliability. With an ICC of 0.855, ${\mathrm{SC}}_{1}$ had a higher reliability than the kyphosis angle of Spine3D [22]. Molinaro et al. noted an intra-day reliability within an ICC range of 0.760 to 0.810 [22]. The lordosis angle was extracted more reliably by Spine3D with an ICC of 0.800 to 0.940 than by torsobarography with an ICC of 0.655. The calculation of associated lordosis angles is causally dependent on the localised lumbar minimum, which also returned a lower reliability compared to the other landmarks. A more consistent localisation of the lumbar landmarks is expected to increase the reliability. Studies using Formetric 4D showed an ICC greater than 0.900 for both the kyphosis angle and the lordosis angle [18,19,20,21]. Considering the sagittal imbalance parameters in comparison to Formetric 4D, it was evident that the ICC values of rasterstereography (ICC = 0.920; [21]) are only slightly higher than those of torsobarography (ICC = 0.868). Nevertheless, the ICC and CV values of the sagittal spine shape parameters showed a high reproducibility when assessing the sagittal imbalance and sagittal posture.

The parameters of commercial systems are mainly limited to the reconstruction of the spine, but the manual clinical examination of posture also includes the symmetry of the anatomical structures like the shoulder girdle, muscles or ribs. This suggests that a posture analysis tool should evaluate the body segments through a wide range of anatomical characteristics [10]. Therefore, the variety of developed thoracic parameters exceeds that of existing commercial systems, offering a broader spectrum for posture analysis.

Shoulder contours as well as scapulae were measured with a good reliability level regarding their vertical position, whereby the ICC values approached the threshold of excellent reliability. The pressure load regions across the scapulae demonstrated a good level of reliability. In contrast, the symmetry comparison of pressure intensities and calculated shifts both exhibited low ICC values. This was potentially linked to the group of subjects predominantly without postural deformities. The SD of shoulders or scapulae localisation was adequate but still too high to detect a small shoulder imbalance. Nevertheless, anatomical structures of the shoulder girdle were localised with almost excellent reproducibility and their characteristics were quantified with a good level of reliability.

The parameters of torso symmetry indicate that higher ICC values for the quantification of pressure intensity in the thoracic region are achieved if the related region is larger. For these body-side-related pressure intensities, ICCs close to and above 0.900 were noted. The resulting symmetry parameters were almost all at a moderate reliability level, in contrast to those of the other parameter groups. In comparison, Molinaro et. al. were able to obtain ICC values in the range of 0.740 to 0.850 for nine male subjects using the torso imbalance parameter of Spine3D [22]. The only parameter that quantified the centre of the pressure curve was at a moderate level, which could provide a reason for a further extended parameterisation. Additional investigations will have to show whether the centre of the pressure curve can be utilised as an alternative to or support the current approximated frontal curve of the spine. For the classification of consistency, the particularly low relative scatter of the parameters describing a ratio of pressure intensities between the left and right torso must be interpreted with caution. The low SD of the parameters combined with an expected mean of 1 for a subject group without pronounced deformities resulted in a low relative scatter. Therefore, it is necessary to identify the threshold value for the severity of a postural deformity above which torsobarography is able to recognise the respective asymmetry.

Currently, none of the commercial systems measure waist asymmetry, although it is considered a clinical indicator of scoliosis. This is the first study to utilise automated waist contour extraction for assessing waist asymmetry. The parameter comparing the shape of the waist contour reached a moderate level and approached a good level of reliability. Accordingly, the parameter may provide an objective assessment method for the severity of waist asymmetry since it scatters in a higher value range when one waist contour is more pronounced than the other.

Sacral parameters, which were supposed to describe the posture of the pelvis, indicated that the level of reliability for localising the pelvic contour was lower than for the other parameter groups. The sagittal slope of the mean pressure values along the lumbosacral transition region was the only parameter that yielded reproducible results with good reliability. Depending on the extraction method, the body-side-related pressure intensities exhibited a good level of reliability. However, according to the ICC, the parameters derived from this to describe the asymmetry tended to have poor reliability. The ratios indicated a rather symmetrical pressure distribution. In general, the reliable objective assessment of pelvic posture is still a major challenge. Similarly insufficient ICCs were reported for rasterstereographic measurement systems: Tabard-Fougère et al. achieved an ICC of 0.500 for the parameter of pelvic obliquity [19]. Degenhardt et al. also achieved rather lower ICC values for the parameters of pelvic torsion (ICC = 0.680) and pelvic rotation (ICC = 0.610) [21].

### 4.2. Limitations and Future Works

The majority of subjects within this study had no diagnosed postural deformity, with the exception of two scoliotic subjects. This was particularly evident for parameters that are intended to indicate an asymmetry between the two sides of the body (e.g., an absolute shift or a ratio). It resulted in a low variability in the respective parameters between the subjects. This in turn led to lower ICC values. The situation differs for parameters that represented the degree of a specific structure, such as kyphosis angle. These parameters varied individually for each subject and tended to exhibit greater variability between subjects, whereby higher ICC values were achieved for such individual parameters.

Lower ICC values were achieved for parameters that describe a symmetry-related postural deformity (e.g., pelvic obliquity with ICC = 0.500 [19] or pelvic rotation with ICC = 0.610 [21]). Despite their poor to moderate reliability, the parameters introduced in this or other studies may still be capable of detecting and assessing postural deformities. It is expected that the respective parameters for a subject population without postural deformities will scatter over a different range than in a subject population with postural deformities. Therefore, statistical validation is required to verify if the parameters for distinct degrees of postural deformity fluctuate in different ranges. Moreover, the mean SDs in the identified localisations imply that postural deformity can only be determined beyond a certain degree of severity.

A structurally rotated torso, as occurs with a rib hump, produces an asymmetrical pressure image. However, it is not yet clear to what extent this asymmetry is transferred onto the pressure image. If both the morphology was transferred linearly to the pressure image and no significant anatomical displacements occurred in the body due to the lying position, established analyses standards from radiological and rasterstereographic measurement methods could be applied to torsobarography. The differences between a standing position and a lying position with respect to posture analysis should be investigated in further studies. The influence of this effect is still unknown. If the moderate reliability of the sacral parameters can be attributed to the variations in the knee angle, an improved method for the reliable positioning of subjects is required.

The parameters consisting of pressure intensities in the lumbar and sacral region showed a lower reproducibility. A knee angle of 100° was confirmed with 5% uncertainty, leading to fluctuations. Bending the knees induces rotation of the hip joint, which in turn leads to the pelvis rotating around the frontal axis. In consequence, this compresses or stretches the lumbar lordosis, leading to fluctuations in the lumbar–sacral region. Furthermore, varying knee angles result in a shift in the body’s centre of mass, which subsequently affects both the pressure intensity and the pressure distribution. Future research should further investigate the effects of lying position on the pressure image, as specified by the standardised measurement procedure.

Further optimisation of the self-inflating pad beneath the sensor mat is necessary. Depending on the elasticity of the underlay, the pressure distribution changes. A soft underlay could possibly emphasise the peripheral areas of the torso, such as the shoulder contours, better. A hard underlay, on the other hand, accentuates prominent structures such as the shoulder blades. Therefore, it is advisable to examine the pad’s transmission characteristics to facilitate the selection of an optimal pad. Such improvements could enhance the detection of the torso and ensure more consistent measurement conditions, which are currently influenced by the varying volume of the self-inflating pad.

## 5. Conclusions

This study introduced torsobarography as an innovative tool for a reproducible analysis of dorsal trunk morphology, demonstrating its potential in postural assessment. Anatomically associated landmarks along the longitudinal axis as well as other morphological structures of the back were quantified with a good to excellent reliability level. The findings suggest that torsobarography may be a competitive alternative to established commercial systems in terms of reproducibility. In particular, the extraction of parameters to describe the sagittal spine shape was highly reliable for individual subjects. The observed low variability among subjects posed challenges to the reliability assessment of the asymmetry parameters, indicating the need for further validation in a more diverse subject pool. Future research is required to ascertain the specific severity threshold at which torsobarography can reliably identify postural deformities. Differences to the standing posture still need to be investigated. Overall, this work paves the way for a new, radiation-free, flexible and easy-to-use tool to detect postural deformities at an early stage or to assess their progression.

## Figures and Tables

**Table 1:** Structure of the parameter extraction in association with diagnostic medical features of postural disorders.

	Specification	Parameter Extraction	Diagnostic Medical Feature
**Spinal** **shape**	Frontal curve	Lateral curvature of the concave canal in the spinal range	Lateral curvature of the spine
	Sagittal curve	Curvature characteristics of the pressure intensities along the longitudinal axis	Imbalance of thoracic kyphosis and lumbar lordosis
**Thoracic** **region**	Shoulder contour	Symmetry of the shoulder contour (position, morphology)	Height difference of the shoulder and shoulder protraction
	Shoulder blades	Symmetry of the accentuated scapula maxima (position, intensity)	Unequal shoulders due to height difference or uneven protrusion
	Torso symmetry *	Symmetry of pressure intensities in the thoracic to lumbar region (intensity, morphology)	Asymmetrical trunk due to rib hump or lumbar prominence
**Lumbar** **region**	Waist contour	Symmetry of the waist contour (position, morphology)	Waist asymmetry
**Sacral** **region**	Sagittal	Slope of the pressure intensities in the lumbar–sacral region	Abnormal pelvic tilt
	Pelvic contour	Positional symmetry of the pelvic contour	Pelvic obliquity
	Pelvic symmetry	Intensity symmetry in the sacral region	Pelvic torsion or pelvic rotation

* Parameters describing torso symmetry use both thoracic and lumbar regions.

**Table 2:** Grand mean calculated by averaging the mean values of the subjects over ten repetitions (Mean), mean standard deviation (SD), mean coefficient of variation (CV), intraclass correlation coefficient (ICC(1,1)) with associated confidence interval (95% CI) for landmarks and reference distances; mean and SD are given in pixels (px) as well as in mm due to the resolution.

Landmarks and Reference Distances	Mean		SD		CV (%)	ICC(1,1)	95% CI
	(px)	(mm)	(px)	(mm)			
Torso start $j_{\mathrm{ts}}$	31.7	161.7	1.9	9.7	6.1	0.882	0.829–0.927
Thoracic regions start $j_{t}$	39.9	203.5	1.6	8.2	4.1	0.917	0.877–0.949
Thoracic maximum $j_{\mathrm{tmax}}$	51.7	263.7	1.6	8.2	3.1	0.913	0.871–0.946
Thoracolumbar transition $j_{\mathrm{tl}}$	72.3	368.7	2.4	12.2	3.3	0.841	0.773–0.899
Lumbar minimum $j_{\mathrm{lmin}}$	93.1	474.8	3.3	16.8	3.5	0.584	0.468–0.708
Lumbar regions end $j_{l}$	111.3	567.6	1.8	9.2	1.6	0.885	0.833–0.928
Sacral maximum $j_{\mathrm{smax}}$	130.2	664.0	2.0	10.2	1.6	0.684	0.580–0.788
Left reference position $i_{l}$	23.9	121.9	0.6	3.1	2.4	0.893	0.844–0.934
Right reference position $i_{r}$	74.5	380.0	0.5	2.6	0.7	0.918	0.880–0.950
Torso length $d_{l}$	99.5	507.5	2.3	11.7	2.3	0.798	0.717–0.870
Torso width $d_{w}$	51.6	263.2	1.1	5.6	2.1	0.911	0.870–0.945
Thoracic region length $d_{\mathrm{th}\;}$	33.4	170.3	1.9	9.7	5.7	0.846	0.780–0.903
Lumbar region length $d_{\mathrm{lu}\;}$	40.0	204.0	2.3	11.7	6.2	0.896	0.848–0.936
Sacral region length $d_{s\;}$	49.7	253.5	1.8	9.2	3.5	0.885	0.833–0.928

**Table 3:** Grand mean calculated by averaging the mean values of the subjects over ten repetitions (Mean), mean standard deviation (SD), mean coefficient of variation (CV), intraclass correlation coefficient (ICC(1,1)) with associated confidence interval (95% CI) for the parameters that approximate the curve of frontal and sagittal spinal shape. Mean and SD are given in pixels (px) as well as in mm due to the resolution; parameters derived from the ratios of pressure intensities are non-dimensional (n. d.). All parameters were referenced to the equations (Eq.) in the previous chapter.

Parameters			Eq.	Mean	SD	CV (%)	ICC(1,1)	95% CI
Frontal spine curve	${\mathrm{FC}}_{1}$	(px)	(8)	2.5	0.7	30.5	0.492	0.374–0.629
		(mm)		12.8	3.6			
	${\mathrm{FC}}_{2}$	(${\mathrm{px}}^{2}$)	(9)	0.4	0.3	69.9	0.572	0.455–0.698
		(${\mathrm{mm}}^{2}$)		10.4	7.0			
	${\mathrm{FC}}_{3}$	(n. d.)	(10)	0.99	0.01	1.3	0.436	0.320–0.577
	${\mathrm{FC}}_{4}$	(px)	(11)	1.1	0.6	62.6	0.458	0.340–0.597
		(mm)		5.6	3.1			
	${\mathrm{FC}}_{5}$	(px)	(12)	1.5	0.5	36.8	0.318	0.212–0.460
		(mm)		7.7	2.6			
	${\mathrm{FC}}_{6}$	(px)	(13)	19.7	8.6	44.7	0.395	0.281–0.538
		(mm)		100.5	43.9			
	${\mathrm{FC}}_{7}$	(n. d.)	(14)	0.02	0.01	60.4	0.449	0.332–0.590
Sagittal spine curve	${\mathrm{SC}}_{1}$	(n. d.)	(15)	1.47	0.21	12.4	0.855	0.793–0.909
	${\mathrm{SC}}_{2}$	(n. d.)	(16)	1.89	0.47	21.3	0.650	0.540–0.761
	${\mathrm{SC}}_{3}$	(n. d.)	(17)	1.40	0.07	4.8	0.838	0.769–0.897
	${\mathrm{SC}}_{4}$	(n. d.)	(18)	1.07	0.12	11.2	0.836	0.766–0.896
	${\mathrm{SC}}_{5}$	(n. d.)	(19)	1.22	0.03	2.6	0.868	0.810–0.917
	${\mathrm{SC}}_{6}$	(n. d.)	(20)	82.18	12.22	15.8	0.842	0.775–0.900
	${\mathrm{SC}}_{7}$	(n. d.)	(21)	1.16	0.02	1.4	0.848	0.783–0.904
	${\mathrm{SC}}_{8}$	(n. d.)	(22)	0.84	0.03	3.7	0.830	0.759–0.892

**Table 4:** Grand mean calculated by averaging the mean values of the subjects over ten repetitions (Mean), mean standard deviation (SD), mean coefficient of variation (CV), intraclass correlation coefficient (ICC(1,1)) with associated confidence interval (95% CI) for the parameters to assess shoulder contour, shoulder blades and torso symmetry. Mean and SD are given in pixels (px) as well as in mm due to the resolution; parameters derived from the ratios of preprocessed pressure values (prv) are non-dimensional (n. d.). All parameters were referenced to the equations (Eq.) in the previous chapter.

Parameters			Eq.	Mean	SD	CV (%)	ICC(1,1)	95% CI
Shoulder contour	$S_{0,l}$	(px)	(23)	31.3	2.1	6.8	0.868	0.809–0.917
		(mm)		159.6	10.7			
	$S_{0,r}$	(px)	(23)	30.5	2.0	6.7	0.866	0.807–0.916
		(mm)		155.6	10.2			
	$S_{1}$	(px)	(23)	1.3	0.8	72.5	0.315	0.209–0.456
		(mm)		6.6	4.1			
	$S_{2}$	(n. d.)	(24)	1.41	0.30	19.1	0.346	0.237–0.489
	$\left|S_{2,l}\right|$	(n. d.)	(24)	0.54	0.09	17.6	0.732	0.636–0.823
	$\left|S_{2,r}\right|$	(n. d.)	(24)	0.46	0.10	22.2	0.664	0.556–0.772
Shoulder blades	${\mathrm{SB}}_{0,\mathrm{vl}}$	(px)	(25)	50.6	2.1	4.2	0.872	0.815–0.920
		(mm)		258.1	10.7			
	${\mathrm{SB}}_{0,\mathrm{vr}}$	(px)	(26)	50.2	1.9	4.0	0.871	0.814–0.919
		(mm)		256.0	9.7			
	${\mathrm{SB}}_{0,\mathrm{hl}}$	(px)	(25)	30.7	1.0	3.1	0.801	0.722–0.872
		(mm)		156.6	5.1			
	${\mathrm{SB}}_{0,\mathrm{hr}}$	(px)	(26)	67.9	1.1	1.7	0.732	0.636–0.823
		(mm)		346.3	5.6			
	${\mathrm{SB}}_{1}$	(px)	(27)	1.9	1.3	78.1	0.345	0.236–0.488
		(mm)		9.7	6.6			
	${\mathrm{SB}}_{2}$	(px)	(28)	1.8	1.3	78.3	0.279	0.178–0.418
		(mm)		9.2	6.6			
	${\mathrm{SB}}_{3}$	(°)	(29)	2.9	2.0	78.4	0.333	0.225–0.475
	${\mathrm{SB}}_{4}$	(n. d.)	(30)	1.05	0.03	2.4	0.478	0.360–0.616
	${\mathrm{SB}}_{4,l}$	(prv)	(30)	1.30 × 10^4^	450	3.4	0.772	0.685–0.852
	${\mathrm{SB}}_{4,r}$	(prv)	(30)	1.27 × 10^4^	367	2.8	0.836	0.767–0.896
	${\mathrm{SB}}_{5}$	(n. d.)	(31)	1.02	0.01	1.2	0.313	0.208–0.454
	${\mathrm{SB}}_{5,l}$	(prv)	(31)	1.15 × 10^4^	186	1.6	0.808	0.731–0.877
	${\mathrm{SB}}_{5,r}$	(prv)	(31)	1.14 × 10^4^	182	1.6	0.834	0.764–0.895
Torso symmetry	${\;\mathrm{TS}}_{1}$	(px)	(36)	50.7	12.3	26.3	0.582	0.466–0.706
		(mm)		258.6	62.7			
	${\;\mathrm{TS}}_{2}$	(n. d.)	(37)	1.03	0.02	1.8	0.384	0.271–0.527
	${\mathrm{TS}}_{2,l}$	(prv)	(37)	8.26 × 10^3^	207	2.6	0.863	0.802–0.914
	${\mathrm{TS}}_{2,r}$	(prv)	(37)	8.23 × 10^3^	198	2.4	0.861	0.800–0.913
	${\;\mathrm{TS}}_{3}$	(n. d.)	(38)	1.02	0.01	0.9	0.592	0.477–0.715
	${\mathrm{TS}}_{3,l}$	(prv)	(38)	1.11 × 10^4^	138	1.2	0.910	0.868–0.945
	${\mathrm{TS}}_{3,r}$	(prv)	(38)	1.10 × 10^4^	146	1.3	0.890	0.840–0.932
	${\;\mathrm{TS}}_{4}$	(n. d.)	(39)	1.25	0.15	11.1	0.564	0.447–0.691
	${\mathrm{TS}}_{4,l}$	(prv)	(39)	6.15 × 10^6^	1.07 × 10^6^	21.2	0.696	0.593–0.796
	${\mathrm{TS}}_{4,r}$	(prv)	(39)	5.96 × 10^6^	1.08 × 10^6^	21.4	0.722	0.624–0.816
	${\;\mathrm{TS}}_{5}$	(%)	(40)	11.5	2.4	22.3	0.561	0.444–0.689

**Table 5:** Grand mean calculated by averaging the mean values of the subjects over ten repetitions (Mean), mean standard deviation (SD), mean coefficient of variation (CV), intraclass correlation coefficient (ICC(1,1)) with associated confidence interval (95% CI) for the waist symmetry assessment parameters based on the extracted waist contour. Mean and SD are sometimes given in pixels (px) as well as in mm due to the resolution; parameters derived from the ratios of pressure intensities are non-dimensional (n. d.). All parameters were referenced to the equations (Eq.) in the previous chapter.

Parameters			Eq.	Mean	SD	CV (%)	ICC(1,1)	95% CI
Waist contour	$W_{0,\mathrm{vl}}$	(px)	(41)	92.5	2.9	3.1	0.664	0.557–0.772
		(mm)		471.8	14.8			
	$W_{0,\mathrm{vr}}$	(px)	(42)	89.8	2.9	3.2	0.688	0.584–0.790
		(mm)		458.0	14.8			
	$W_{0,\mathrm{hl}}$	(px)	(41)	18.8	0.8	4.7	0.864	0.804–0.915
		(mm)		95.9	4.1			
	$W_{0,\mathrm{hr}}$	(px)	(42)	17.5	0.8	4.8	0.871	0.814–0.919
		(mm)		89.3	4.1			
	$W_{1}$	(px)	(43)	3.4	2.2	67.4	0.390	0.277–0.533
		(mm)		17.3	11.2			
	$W_{2}$	(px)	(44)	1.5	0.9	64.9	0.229	0.137–0.361
		(mm)		7.7	4.6			
	$W_{3}$	(°)	(45)	5.4	3.3	66.7	0.425	0.309–0.567
	$W_{4}$	(n. d.)	(46)	1.35	0.21	14.6	0.689	0.585–0.791
	$\left|W_{4,l}\right|$	(n. d.)	(46)	1.08 × 10^−2^	1.93 × 10^−3^	18.9	0.733	0.637–0.824
	$\left|W_{4,r}\right|$	(n. d.)	(46)	1.00 × 10^−2^	1.75 × 10^−3^	18.2	0.726	0.629–0.819

**Table 6:** Grand mean calculated by averaging the mean values of the subjects over ten repetitions (Mean), mean standard deviation (SD), mean coefficient of variation (CV), intraclass correlation coefficient (ICC(1,1)) with associated confidence interval (95% CI) for parameters describing the pelvic posture located in the sacral region. Mean and SD are sometimes given in pixels (px) as well as in mm due to the resolution; parameters derived from the ratios of preprocessed pressure values (prv) are non-dimensional (n. d.). All parameters were referenced to the equations (Eq.) in the previous chapter.

Parameters			Eq.	Mean	SD	CV (%)	ICC(1,1)	95% CI
Pelvic posture	$P_{0,\mathrm{ul}}$	(px)	(47)	117.3	2.3	2.0	0.733	0.637–0.824
		(mm)		598.2	11.7			
	$P_{0,\mathrm{ur}}$	(px)	(48)	117.0	2.3	2.0	0.747	0.654–0.834
		(mm)		596.7	11.7			
	$P_{0,\mathrm{ll}}$	(px)	(47)	148.7	1.8	1.2	0.571	0.454–0.697
		(mm)		758.4	9.2			
	$P_{0,\mathrm{lr}}$	(px)	(48)	148.5	1.7	1.1	0.665	0.558–0.773
		(mm)		757.4	8.7			
	$P_{1}$	(prv/px)	(49)	120.6	17.8	15.4	0.771	0.684–0.851
	$P_{2}$	(px)	(50)	2.5	1.5	62.2	0.212	0.124–0.342
		(mm)		12.8	7.7			
	$P_{3}$	(n. d.)	(51)	1.13	0.09	8.1	0.203	0.116–0.331
	$P_{3,l}$	(prv)	(51)	5.11 × 10^6^	7.78 × 10^5^	15.3	0.608	0.494–0.728
	$P_{3,r}$	(prv)	(51)	4.81 × 10^6^	7.33 × 10^5^	15.4	0.651	0.542–0.762
	$P_{4}$	(n. d.)	(52)	1.01	3.65 × 10^5^	0.4	0.284	0.183–0.423
	$P_{4,l}$	(prv)	(52)	1.13 × 10^4^	80	0.7	0.873	0.816–0.920
	$P_{4,r}$	(prv)	(52)	1.13 × 10^4^	83	0.7	0.858	0.796–0.911

## Data Availability

The data presented in this study are available on reasonable request from the corresponding author.
